# Reduced Uterine Perfusion Pressure (RUPP) Model of Preeclampsia in Mice

**DOI:** 10.1371/journal.pone.0155426

**Published:** 2016-05-17

**Authors:** Tomofumi Fushima, Akiyo Sekimoto, Takahiro Minato, Takuya Ito, Yuji Oe, Kiyomi Kisu, Emiko Sato, Kenichi Funamoto, Toshiyuki Hayase, Yoshitaka Kimura, Sadayoshi Ito, Hiroshi Sato, Nobuyuki Takahashi

**Affiliations:** 1 Division of Clinical Pharmacology and Therapeutics, Tohoku University Graduate School of Pharmaceutical Sciences and Faculty of Pharmaceutical Sciences, Sendai, Japan; 2 Advanced Interdisciplinary Biomedical Engineering, Tohoku University Graduate School of Medicine, Sendai, Japan; 3 Division of Nephrology, Endocrinology, and Vascular Medicine, Tohoku University Graduate School of Medicine, Sendai, Japan; 4 Japan Frontier Research Institute for Interdisciplinary Sciences, Tohoku University, Sendai, Japan; 5 Institute of Fluid Science, Tohoku University, Sendai, Japan; Michigan State University, UNITED STATES

## Abstract

Preeclampsia (PE) is a pregnancy-induced hypertension with proteinuria that typically develops after 20 weeks of gestation. A reduction in uterine blood flow causes placental ischemia and placental release of anti-angiogenic factors such as sFlt-1 followed by PE. Although the reduced uterine perfusion pressure (RUPP) model is widely used in rats, investigating the role of genes on PE using genetically engineered animals has been problematic because it has been difficult to make a useful RUPP model in mice. To establish a RUPP model of PE in mice, we bilaterally ligated ovarian vessels distal to ovarian branches, uterine vessels, or both in ICR-strain mice at 14.5 days post coitum (dpc). Consequently, these mice had elevated BP, increased urinary albumin excretion, severe endotheliosis, and mesangial expansion. They also had an increased incidence of miscarriage and premature delivery. Embryonic weight at 18.5 dpc was significantly lower than that in sham mice. The closer to the ligation site the embryos were, the higher the resorption rate and the lower the embryonic weight. The phenotype was more severe in the order of ligation at the ovarian vessels < uterine vessels < both. Unlike the RUPP models described in the literature, this model did not constrict the abdominal aorta, which allowed BP to be measured with a tail cuff. This novel RUPP model in mice should be useful for investigating the pathogenesis of PE in genetically engineered mice and for evaluating new therapies for PE.

## Introduction

Preeclampsia (PE) is a pregnancy-induced hypertension with proteinuria that typically occurs after 20 weeks of gestation [1]. PE is a complication of approximately 5% of human pregnancies [2] and can cause maternal death [3]. Although the etiology of PE is not fully understood, it is widely accepted that placental ischemia/hypoxia increases the production by the placenta of soluble fms-like tyrosine kinase (sFlt-1), soluble endoglin, and likely other factors [4]. sFlt-1 is a splice variant of vascular endothelial growth factor (VEGF) receptor 1 that lacks the cytoplasmic and transmembrane domains and is secreted into the circulation. Because sFlt-1 retains the ligand-binding domain [5], it works as an antagonist of VEGF and placental growth factor (PlGF). Both hypertension and proteinuria in PE are experimentally induced by excess sFlt-1 in nonpregnant and pregnant rats [4, 6].

**Fig 1:**
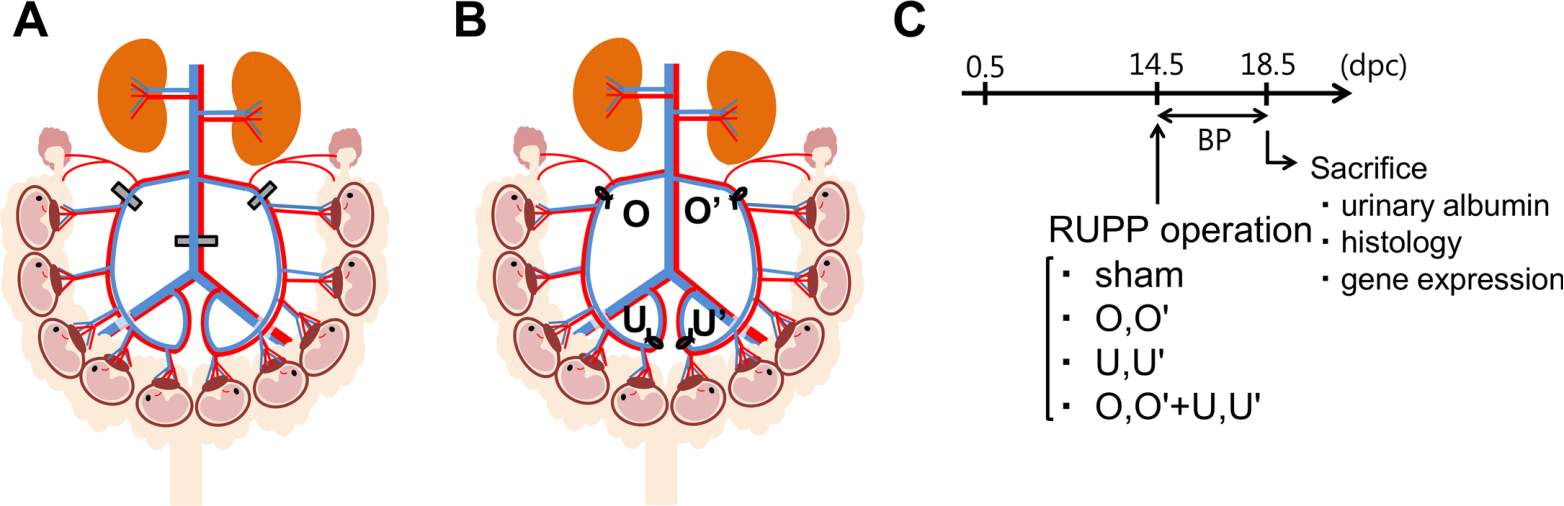
Comparison of conventional reduced uterine perfusion pressure (RUPP) model and novel RUPP model. (**A**) Conventional RUPP model in rats. Silver clips are placed around the aorta right above the iliac bifurcation and around the left and right uterine arcade at the ovarian artery before the first segmental artery. (**B**) Novel RUPP model in mice. At 14.5 dpc, a midabdominal incision was made and a ligature was tied around the uterine arterial and venous branches of the vascular arcade at either ovarian vessels (O,O’) or uterine vessels (U,U’). (**C**) Experimental design.

The reduced uterine perfusion pressure (RUPP) model is widely used in various pregnant animals like rats [7, 8] and baboons [9], but with little success in mice [10]. The rat RUPP model is made by clipping the ovarian arteries and abdominal aorta with silver clips, as described in Fig 1A [11, 12]. The RUPP rat model mimics the physiological features of PE in humans, including hypertension, proteinuria, and fetal growth restriction, with decreased litter size and pup weight [13, 14]. However, in this rat model, tail-cuff blood pressure (BP) is not informative and glomerular endotheliosis does not develop [15]. If mouse RUPP model becomes available, it should serve as a useful tool to advance our understanding of the role of genes on PE using numerous already available genetically engineered mice. Therefore, in the present study, our objective was to develop a useful PE model using an improved RUPP method in mice.

## Materials and Methods

### Animals

All animal experiments were conducted in accordance with the guidelines of Tohoku University. Tohoku University Institutional Animal Care and Use Committee approved this study (2014-014-1). Mice of the ICR strain were purchased from CLEA Japan, Inc. (Tokyo, Japan). Animals were housed individually at 24°C on a 12:12 h light–dark cycle and fed a regular chow and water at libitum. We monitored the body weight and activity of animals at least once a day. When animals lose body weight more than 25% in a few days or look lethargic or dehydrated, we dislocate their neck after they are deeply anesthetized with isoflurane. Embryos were decapitated to euthanize. Complete ligation of both ovarian and uterine vessels killed 4 dams within a day or two after the operation. But all the animals did not look sick. The cause of death was unclear, but their uteri were necrotic. We used buprenorphine (0.05 mg/kg i.p.) at the time of surgery.

### RUPP operation

At the age of 2–3 months, female mice were pair-housed with male mice. The presence of a vaginal plug was designated as 0.5 days postcoitum (dpc). At 14.5 dpc, pregnant mice were anesthetized with isoflurane and a midabdominal incision was made. The number of live and dead embryos was counted through the uterine wall. The ligature was tied around the arterial and venous branches of the uterine vascular arcade. To test the positional effects of ligation of the vascular arcade, we divided the mice into four groups, as shown in Fig 1B and 1C. Sham mice were operated on in a fashion similar to that performed in RUPP mice, but without ligation. In O,O’ mice, ligations were placed around ovarian vessels distal to the branches to the ovaries. In U,U’ mice, ligations were placed around the uterine vessels. In O,O’+U,U’ mice, ligations were placed around both ovarian and uterine vessels. Because complete ligation of both ovarian and uterine vessels killed dams within a day or two after the operation, uterine arteries in O,O’+U,U’ mice were tied with a nylon thread 0.35 mm in diameter, followed by removal of the thread to provide a small space. The abdominal incision was sutured, and mice were given 1mL of oral rehydration solution (0.3% NaCl and 4% sucrose).

### Doppler ultrasonography

Pregnant ICR mice at 14.5 dpc were anesthetized with 2% isoflurane and placed on a heated platform. A midabdominal incision was made, and the arteries of the uterine arcades were observed by an MS550S 40 MHz transducer and Vevo® 2100 ultrasound system (Visualsonics, Toronto, ON, Canada) as we and other investigators described previously [16, 17]. Doppler waveforms were obtained before and immediately after ligating uterine or ovarian vessels. Peak systolic velocity parallel to the arterial blood flow was measured from four consecutive cardiac cycles in each artery.

### Measurement of BP

Because our preliminary study revealed that radio-telemetry method of measuring BP is stressful, and increases the incidence of miscarriage in pregnant mice, BP was noninvasively measured daily (between 10 am to noon) from 14.5 to 18.5 dpc by determining the tail blood volume with a volume pressure recording sensor and an occlusion tail cuff (CODA System; Kent Scientific, Torrington, CT, USA) (Fig 1C) [18, 19].

### Harvesting samples

As shown in Fig 1C, blood was collected at 18.5 dpc. Housing pregnant mice in metabolic cages for urine collection increased BP [20] and the incidence of miscarriage. The volume of 24 hr urine from pregnant mouse is very small, and it is difficult to collect urine samples without food contamination using metabolic cages. Depriving food from pregnant mice to avoid food contamination in the urine for extended period of time is not ethical. Accordingly, spot urine samples were collected at 18.5 dpc. Samples were snap frozen in liquid nitrogen and stored at −28°C until assay. Embryos and placentae were removed from the uterus and weighed. Samples for gene expression analysis were collected, snap frozen in liquid nitrogen, and stored at −80°C until assay.

### Biochemical measurement of blood and urine samples

Urinary albumin concentrations were determined with the Mouse Albumin ELISA Quantitation set (Bethyl Laboratories, Montgomery, TX, USA). Plasma concentrations of sFlt-1 and VEGF were determined with ELISA kits from R&D Systems Inc. (Minneapolis, MS, USA). Plasma aspartate aminotransferase (AST) was measured with the Transaminase CII-test kit (Wako Pure Chemicals, Osaka, Japan).

### Morphometric studies

Paraformaldehyde-fixed kidneys were embedded in paraffin, sectioned at a thickness of 2 μm, and stained with periodic acid–Schiff stain. We evaluated endotheliosis by a decrease in glomerular open capillary area expressed as percentages of the glomerular tuft area [18, 19]. Glomerular mesangial expansion is also a measure of glomerular changes in preeclampsia, and was expressed as PAS-positive mesangial area normalized to glomerular tuft area [18, 19]. Thirty randomly selected glomeruli from each mouse were analyzed with Image J.

### Quantitative reverse transcription PCR

RNA was extracted from kidneys and placentae with TRIzol (Invitrogen, Carlsbad, CA, USA). Aliquots (5 μg) of RNA were reverse transcribed with iScript advanced cDNA Synthesis Kit (BioRad, Hercules, CA, USA). Gene expressions were quantified with SsoAdvanced Universal Probes Supermix (BioRad, Hercules, CA), with hypoxanthine-guanine phosphoribosyl transferase (*Hprt*) as a reference gene [19].

### Statistical analysis

Data were analyzed by Student’s *t*-test or Wilcoxon test between two groups. Analysis of variance (ANOVA) with Dunnett’s test or Tukey–Kramer test was used for comparisons among three or more groups. All analyses were performed with the program JMP 11 (SAS Institute Inc., Cary, NC, USA). Values were presented as means ± standard error of the mean (SEM). A *p* value of < 0.05 was considered statistically significant.

## Results

### Ligation of ovarian or uterine vessels reduces arterial blood flow in uterine arcade

**Fig 2:**
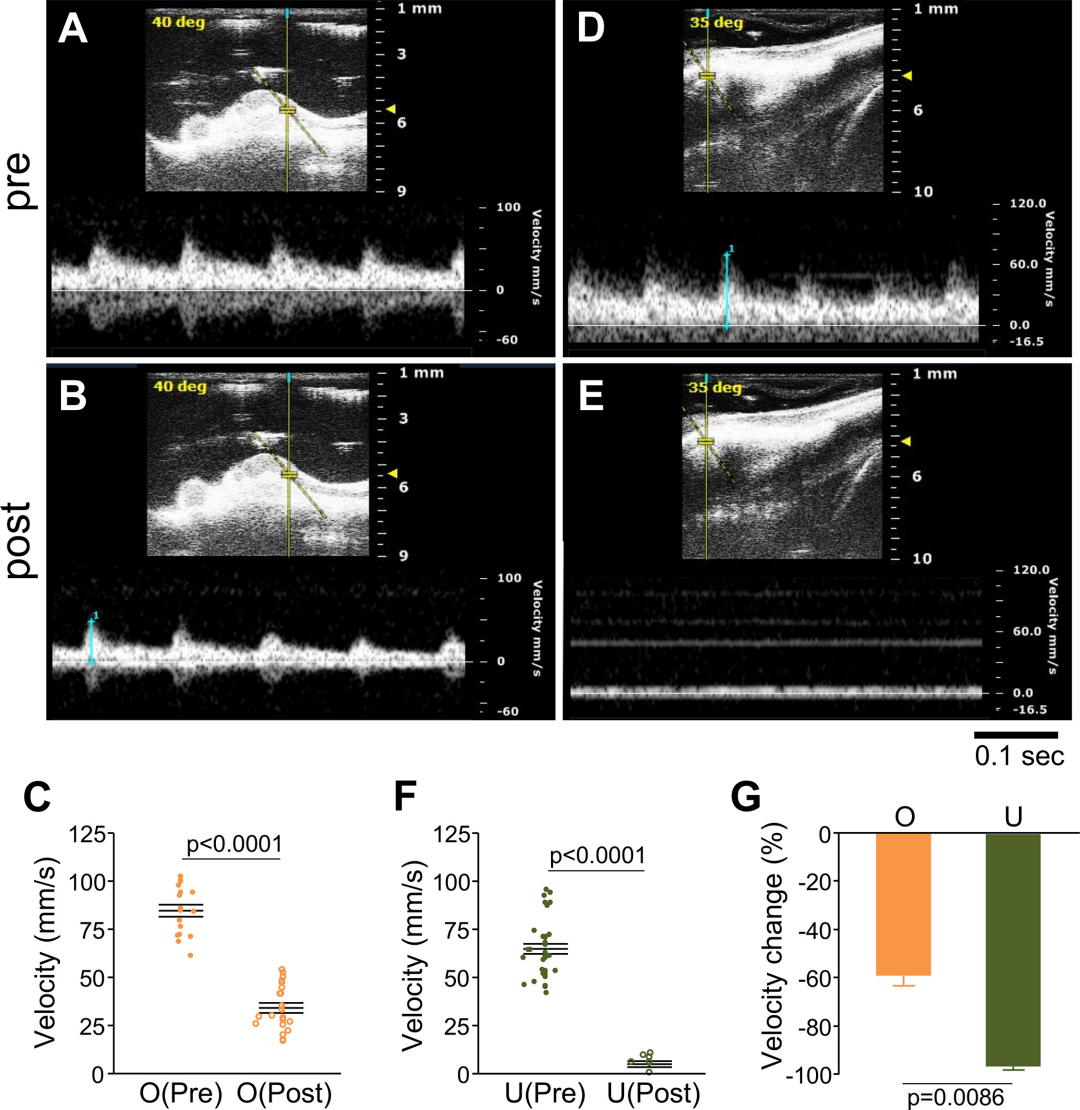
Ligation of uterine vessels reduces arterial blood flow of uterine arcades. Doppler blood velocity waveforms were obtained from an artery of the uterine arcade before (**A**) or after (**B**) ligation of ovarian vessels. (**C**) Summary of arterial blood flow velocity of 4 uterine arcades (4 peaks each) before and after ligation of ovarian vessels. Doppler blood velocity waveforms were obtained from an artery of the uterine arcade before (**D**) or after (**E**) ligation of uterine vessels. (**F**) Summary of arterial blood flow velocity of 5 uterine arcades (4 peaks each) before and after ligation of uterine vessels. (**G**) Percentage of velocity change relative to velocity before ligation. n = 4 (ovarian vessels) and n = 5 (uterine vessels).

Because the uterus is supplied with blood from both the ovarian and uterine arteries [21], we first compared the influence on uterine blood flow of ligating the ovarian (O,O’) and uterine (U,U’) vessels. Ligation of unilateral ovarian vessels (O) caused a 60% decline in the arterial blood flow velocity (from 84.7 ± 3.1 mm/s to 34.4 ± 2.6 mm/s, *p* < 0.0001) without increasing the pulse rate (Fig 2A–2C). Ligation of unilateral uterine vessels (U) caused a 90% decline in the arterial blood flow velocity (from 65.2 ± 2.5 mm/s to 5.3 ± 1.4 mm/s, *p* < 0.0001) (Fig 2D–2F). U ligation decreased the uterine arterial blood flow velocity greater than O ligation (*p* < 0.0001) (Fig 2G). When uterine vessels were tied with nylon thread, followed by removal of the thread to provide a small space, the arterial blood flow velocity partially recovered (from 68.3 ± 2.7 mm/s to 10.0 ± 2.3 mm/s to 26.5 ± 1.8 mm/s) (Fig D in S1 Fig). These results indicate that the uterine arteries play a more dominant role in blood supply to the uterus compared to the ovarian arteries in our experimental condition.

### Hypertension, proteinuria, and endotheliosis in RUPP mice

The basic characteristics of the RUPP mice are shown in S1 Table. Body weights of the dams did not differ among the four groups until surgery at 14.5 dpc, but those of U,U’ and O,O’+U,U’ mice decreased after the surgery. At 18.5 dpc, they were significantly lower than those of sham mice (59.5 ± 1.6 g). Kidney weights were similar among the four groups. The spleens of U,U’ and O,O’+U,U’ mice were heavier than those of sham mice, although this difference was not statistically significant. The hearts of U,U’ and O,O’+U,U’ mice were significantly heavier than those of sham mice.

**Fig 3:**
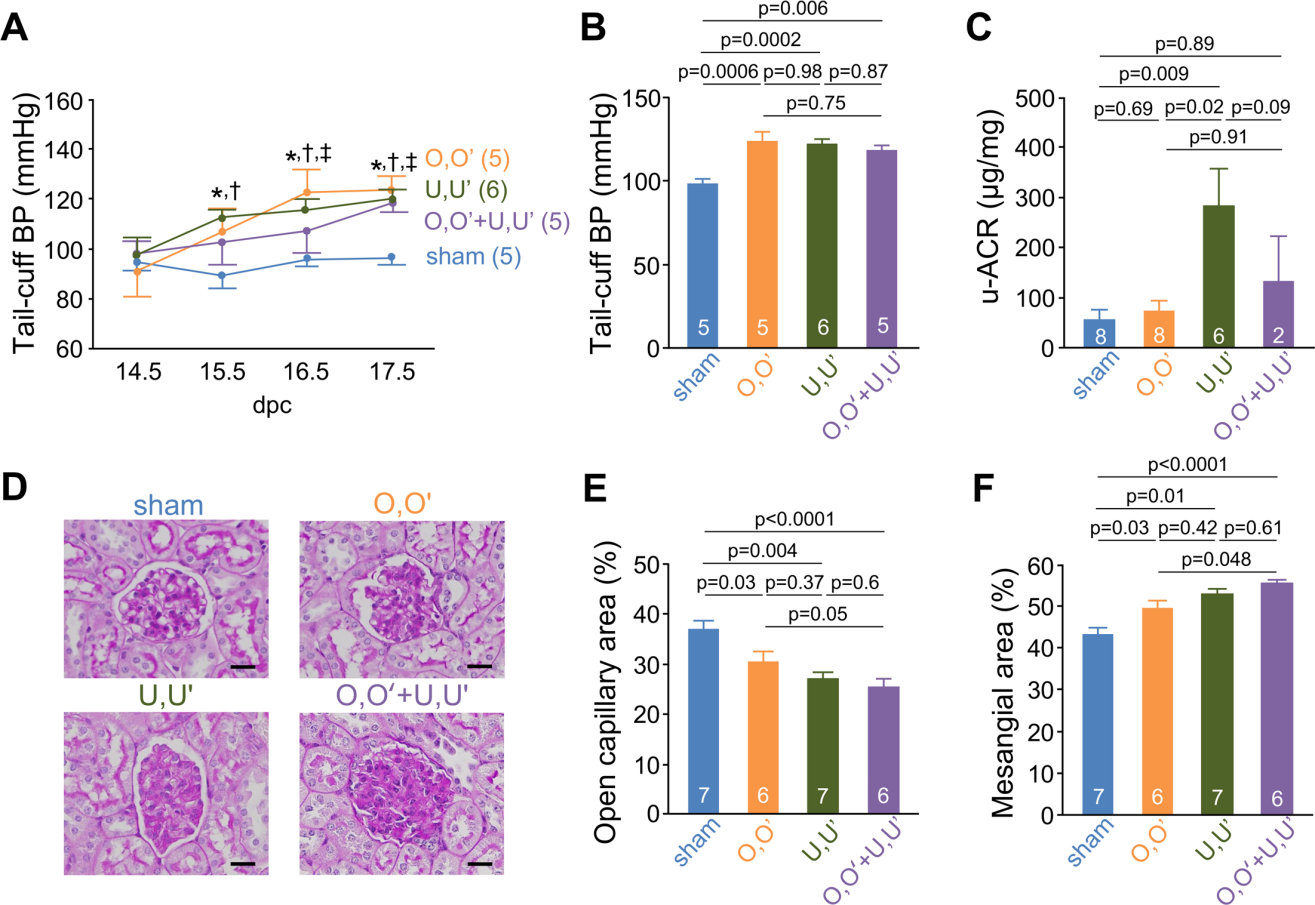
RUPP causes hypertension, proteinuria, and glomerular endotheliosis. (**A**) Tail-cuff blood pressure. *p < 0.05 between sham and O,O’; †*p* < 0.05 between sham and U,U’; ‡p<0.05 between sham and O,O’+U,U’. (**B**) Tail-cuff blood pressure at 17.5 dpc, 1 day before animals were killed. (**C**) Albumin/creatinine ratio (ACR) of urine samples at 18.5 dpc. (**D**) Representative glomeruli from sham, O,O’, U,U’, and O,O’+U,U’. Periodic acid–Schiff stain. Bars, 20 μm. (**E**), (**F**) Quantitative estimates of glomerular open capillary area (index of endotheliosis) (**E**) and PAS-positive area (mesangium) (**F**) in the kidneys of 18.5 dpc pregnant mice normalized to glomerular tuft area.

BP was significantly elevated as early as 1 day after the operation (Fig 3A). At 17.5 dpc, RUPP mice maintained a significantly higher BP relative to sham mice (O,O’: 124.2 ± 5.5 mmHg, U,U’: 120.6 ± 3.7 mmHg, O,O’+U,U’: 118.5 ± 2.7 mmHg vs. sham: 96.8 ± 1.9 mmHg) (Fig 3B). The albumin-creatinine ratio (ACR) measured from spot urine collected at 18.5 dpc was significantly higher in U,U’ mice (299 ± 113 μg/mg) than in sham mice (53 ± 21 μg/mg) (Fig 3C).

Glomerular endotheliosis is a characteristic feature of preeclampsia [22]. Light microscopy showed that RUPP mice had endotheliosis, as indicated by the decrease in glomerular open capillary area (O,O’: 30.5%, U,U’: 27.1%, O,O’+U,U’: 24.5% vs. sham: 36.8%) (Fig 3D and 3E). RUPP mice also showed glomerular mesangial expansion (Fig 3D and 3F). These results suggest that the uterine arteries play a more important role in the development of PE than ovarian arteries, which is consistent with their role in blood supply to the uterus.

### Pregnancy outcome in RUPP mice

**Fig 4:**
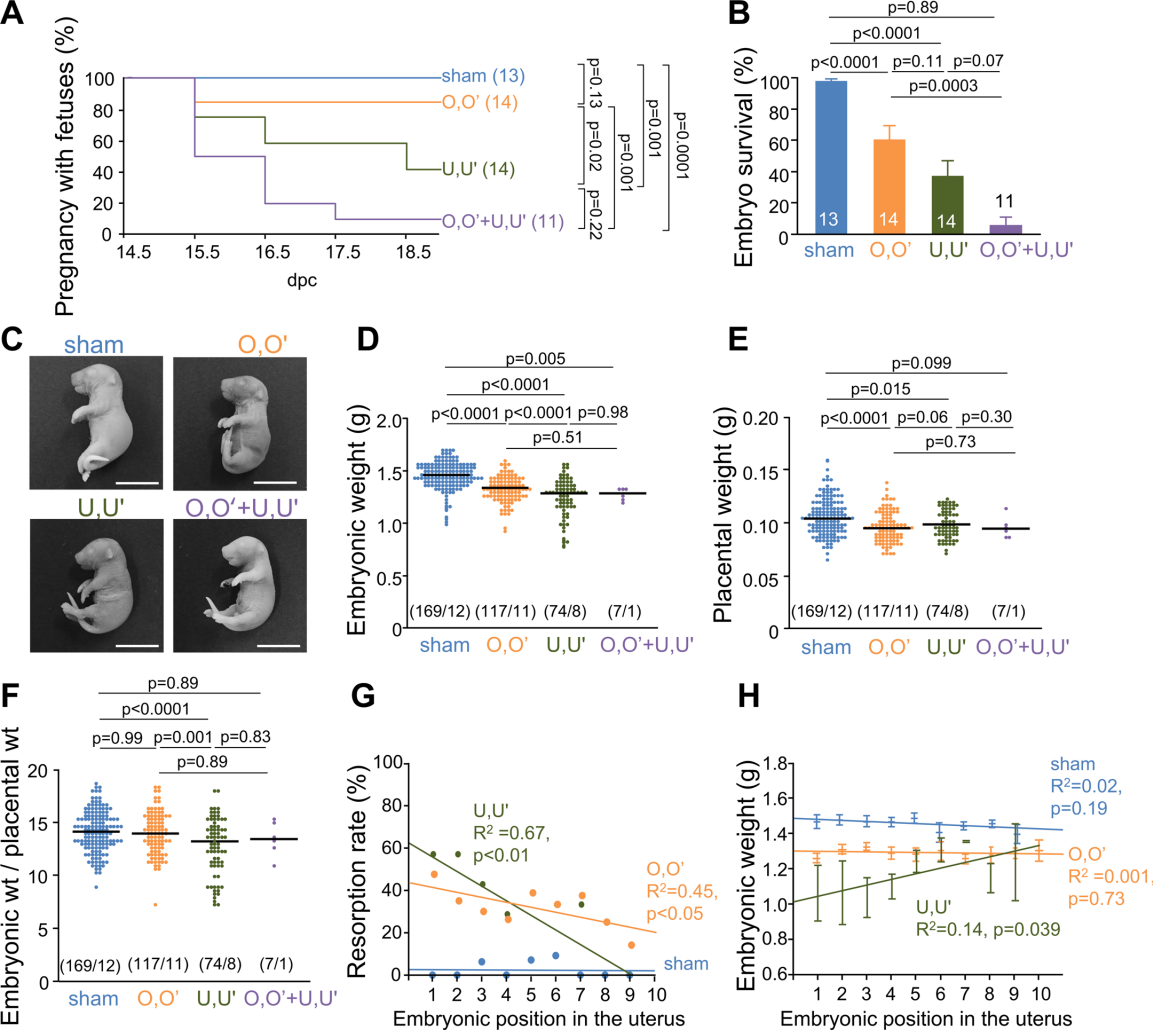
RUPP surgery causes miscarriages and embryonic growth restriction. (**A**) Continuing pregnancies with or without the ligation at 14.5 dpc; parentheses show number of pregnancies at 14.5 dpc. (**B**) Embryo survival from 14.5 dpc to 18.5 dpc. (**C**) Representative images of embryos. Bars, 1 cm. (**D**) Embryonic weight at 18.5 dpc; parentheses show number of embryos/number of dams. (**E**) Placental weight at 18.5 dpc. (**F**) Embryonic weight/placental weight ratios. (**G**) Relationship between resorption rate and embryonic position in the uterus. (**H**) Relationship between embryonic weight and position in the uterus. Embryonic position was numbered from the position of ligation.

Continuing pregnancies were significantly reduced in the ligation group (Fig 4A). As shown in S2 Table, litter size at surgery did not differ among the four groups; however, at 18.5 dpc it was significantly smaller in RUPP mice (O,O’: 8.7 ± 1.2 pups, U,U’: 3.4 ± 1.5 pups, O,O’+U,U’, 1.2 ± 1.8 pups) compared to sham mice (13.4 ± 1.5 pups). The results were similar for embryo survival (Fig 4B). The representative embryos from each group are shown in Fig 4C. Embryonic weight at 18.5 dpc was significantly lower in RUPP mice (O,O’: 1.34 ± 0.01 g, U,U’: 1.25 ± 0.02 g, O,O’+U,U’: 1.27 ± 0.02 g) relative to sham mice (1.45 ± 0.01 g, Fig 4D). There was a similar trend for placental weight, but the changes were smaller (Fig 4E). Moreover, the embryonic weight/placental weight ratio was smaller in the U,U’ mice compared to the other groups of mice (Fig 4F).

In addition to embryonic weight, we also examined the embryonic death rate and weight associated with different positions in the uterus determined as shown in Figs A-C in S2 Fig. Fig 4G and 4H shows resorption rates and embryonic weights plotted against the position in the uterus counted from the ligation points. Although the embryonic position in the uterus did not appear to be associated with resorption and embryonic weight in sham mice, the closer the embryonic position to the ligation point, the higher the resorption rate and the lower the embryonic weight. The slopes of the regression lines in Fig 4G and 4H were steeper for U,U’ mice than for O,O’ mice. Thus, uterine arteries appear to play a more dominant role in maintaining pregnancy and embryonic growth than ovarian arteries.

### sFlt-1 and VEGF levels in the plasma and placenta

**Fig 5:**
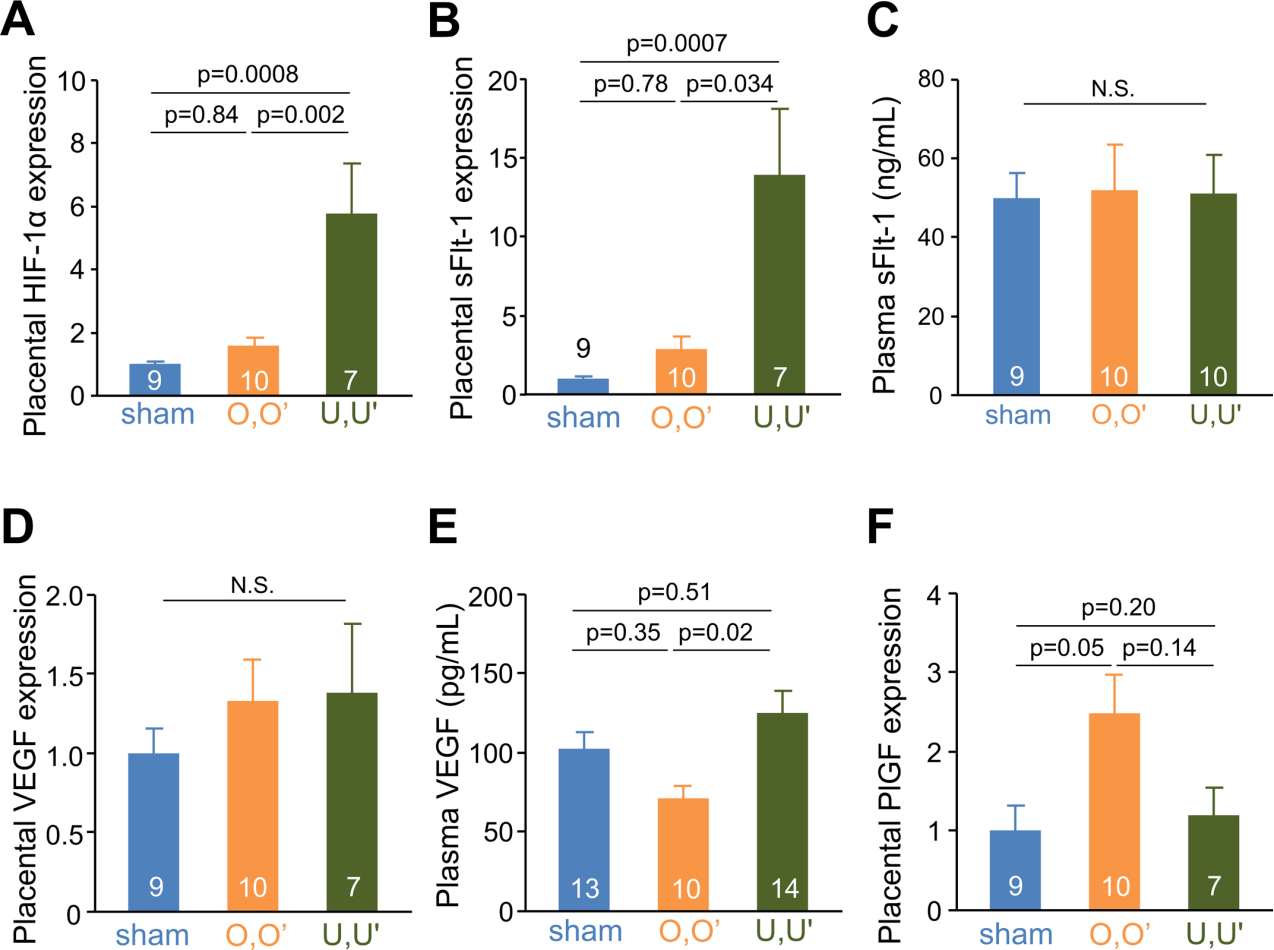
Hypoxia by RUPP increases placental sFlt-1 expression. mRNA expression of HIF-1α (**A**), sFlt-1 (**B**), VEGF (**D**), and PlGF (**F**) in placentae at 18.5 dpc. Plasma concentrations of sFlt-1 (**C**) and VEGF (**E**) at 18.5 dpc dams.

Because increased sFlt-1 production from the ischemic placenta is a common finding in PE, we determined the expression levels of hypoxia inducible factor (HIF-1α) and sFlt-1 mRNA in the placenta. HIF-1α and sFlt-1 mRNA levels were 2.8 times and 9.5 times higher, respectively, in U,U’ mice compared to sham mice (Fig 5A and 5B). However, plasma sFlt-1 concentrations were not elevated in RUPP mice (Fig 5C). RUPP did not affect placental expression of vascular growth factor (VEGF) mRNA and plasma VEGF levels (Fig 5D and 5E). The expression levels of placental growth factor (PlGF) mRNA in the placenta in O,O’ mice were 2.4 times higher compared to sham mice, but not elevated in U,U’ mice (Fig 5F). Expression levels in the placenta of inflammatory cytokine interleukin-6 (IL-6) were not significantly affected by RUPP operation (data not shown).

## Discussion

The present study investigated a novel RUPP model in mice. Like human patients with PE, reduced perfusion of the uterus in mice causes placental sFlt-1 overproduction, hypertension, albuminuria, miscarriage, and fetal growth restriction. Ligation of uterine vessels (U,U’) in ICR mice causes endotheliosis similar to that observed in human patients with PE. Because our RUPP surgery does not touch the abdominal aorta, it is possible to obtain useful BP information with tail cuffs. Ligating ovarian vessels, uterine vessels, and both similarly increased BP (Fig 3A and 3B). Although endotheliosis in preeclampsia has been focused in arteries and glomerular capillaries, elevated sFlt-1 and subsequent decrease in VEGF should cause endothelial damage throughout the blood vessels including veins. Reduced venous capacitance and compliance have been suggested to cause increased BP in PE [23]. Moreover, human umbilical vein endothelial cells have been widely used for investigating endothelial damage in PE. Accordingly, although we ligated both arteries and veins to the uterus for technical reasons, this method of ligation could provide a better model for unraveling pathophysiology of PE than a conventional model induced by clipping only arteries.

Proteinuria was seen in U,U’ mice but not in O,O’ mice (Fig 3C). Urinary albumin excretion was not as high in the O,O’+U,U’ group, probably because those developed more severe PE miscarried before obtaining urine samples. Intapad et al. reported that the conventional RUPP model in C57BL/6J mice did not produce proteinuria [10]. ICR mice could be more prone than C57BL/6J mice to developing proteinuria as a consequence of RUPP. U,U’ mice also showed glomerular mesangial expansion as well as endotheliosis (Fig 3D–3F).

Maintenance of pregnancy deteriorates with decreased uterine blood flow (Fig 4A and 4B). The large number of fetal deaths in our mouse RUPP model is consistent with the results from rat model of RUPP [24]. Fetal growth restriction is a consequence of reduced placental blood flow that is the result of damage to the placental vasculature caused by anti-angiogenic factors and/or to impaired development of the placenta [25]. Fetal growth restriction is frequently seen in preeclamptic women [26, 27]. Consistent with these previous findings, our study showed that ligation of the uterine vessels caused significant embryonic death and decreased embryonic weight. Although reduced blood flow decreased both embryonic and placental weight (Fig 4D and 4E), the effect of blood flow on embryonic growth was larger when uterine vessels were ligated (U,U’) compared to when ovarian vessels were ligated (O,O’), based on the embryonic/placental weight ratio. This ratio was higher than expected in the O,O’+U,U’ group. This is probably because more of the embryos were miscarried in this group than in the other groups. If inspected earlier than 18.5 dpc, one might have been able to see a larger decrease in this group compared to the other three groups.

PE is thought to be caused by a reduction in uterine blood flow and release of sFlt-1 from ischemic and hypoxic placenta [28]. Consistent with the larger decrease in uterine blood flow as a consequence of ligation of uterine vessels compared to ovarian vessels, placentae of U,U’ mice had a greater increase in HIF-1α and sFlt-1 mRNA expression. However, plasma sFlt-1 levels did not differ between the sham and RUPP groups. This is probably because the number of placentae producing sFlt-1 was smaller due to miscarriages and resorption of embryos in RUPP mice, even though each placenta produced more sFlt-1.

Because our RUPP surgery was performed at 14.5 dpc, as in most of the rat RUPP models, when placentation is already finished [29], this model does not address questions about early alterations in the immune system, trophoblast invasion, or placentation. Moreover, although U,U’ mice showed a slight increase in liver enzyme levels, their blood platelet counts did not decrease. This suggests that this model, in its current state, does not develop severe PE as HELLP (hemolysis, elevated liver enzymes, and low platelets) syndrome. However, in combination with genetically engineered mice, this mouse model is expected to serve as a good tool to study the mechanisms and treatment of severe PE and its associated conditions.

In conclusion, we developed a novel RUPP model of PE in mice that causes hypertension, proteinuria, glomerular endotheliosis, fetal growth restriction, and fetal death. Notably, these adverse fetal outcomes were associated with the degree of ischemia. This model recapitulates the human PE phenotype and will be useful for unraveling the pathogenesis of PE using genetically engineered mice and for evaluating new therapies for PE.

## Supporting Information

S1 Fig When a uterine artery was tied with nylon thread, followed by removal of the thread to provide a small space, the arterial blood flow velocity partially recovered. Doppler blood velocity waveforms were obtained from a uterine artery before **(A)** and after **(B)** ligation of uterine vessels with a nylon thread. **(C)** Doppler blood velocity waveforms after removal of the nylon thread. (**D**) Summary of arterial blood flow velocity of 2 uterine arcades (4 peaks each) before ligation, after ligation, and after removal of a nylon thread of uterine vessels.
(TIF)

S2 Fig Numbering embryonic position in the uterus. (**A**) sham mice, (**B**) O,O’ mice, (**C**) U,U’ mice. The number of the embryos were counted from the one closest to the ovaries in sham (panel A) or from the ligation position (panels B and C), when the pregnant mice have 5 embryos in right and left uterine horns each. In reality the number of embryos in each horn is from zero to 9 in ICR strain of mice.
(TIF)

S1 Table Basic characteristics of RUPP mice. ^a^*p* < 0.05 vs. sham, ^b^*p* < 0.05 vs. O,O’, ^c^*p* < 0.05 vs. U,U’. Data are expressed as means ± standard error of the mean. Values in parentheses show body weight and the number of dams without miscarriages at 18.5 dpc.
(PDF)

S2 Table Pregnancy outcome of RUPP mice. ^a^*p* < 0.05 vs. sham, ^b^*p* < 0.05 vs. O,O’, ^c^*p* < 0.05 vs. U,U’, ^d^embryonic weight less than tenth percentile of sham. Data are expressed as means ± standard error of the mean. FGR, fetal growth restriction.
(PDF)
