# Supplementary figures and images for: Reduced Uterine Perfusion Pressure (RUPP) Model of Preeclampsia in Mice
Source: PLoS One. 2016 May 17;11(5):e0155426. doi: 10.1371/journal.pone.0155426 (PMC4871336; doi:10.1371/journal.pone.0155426)

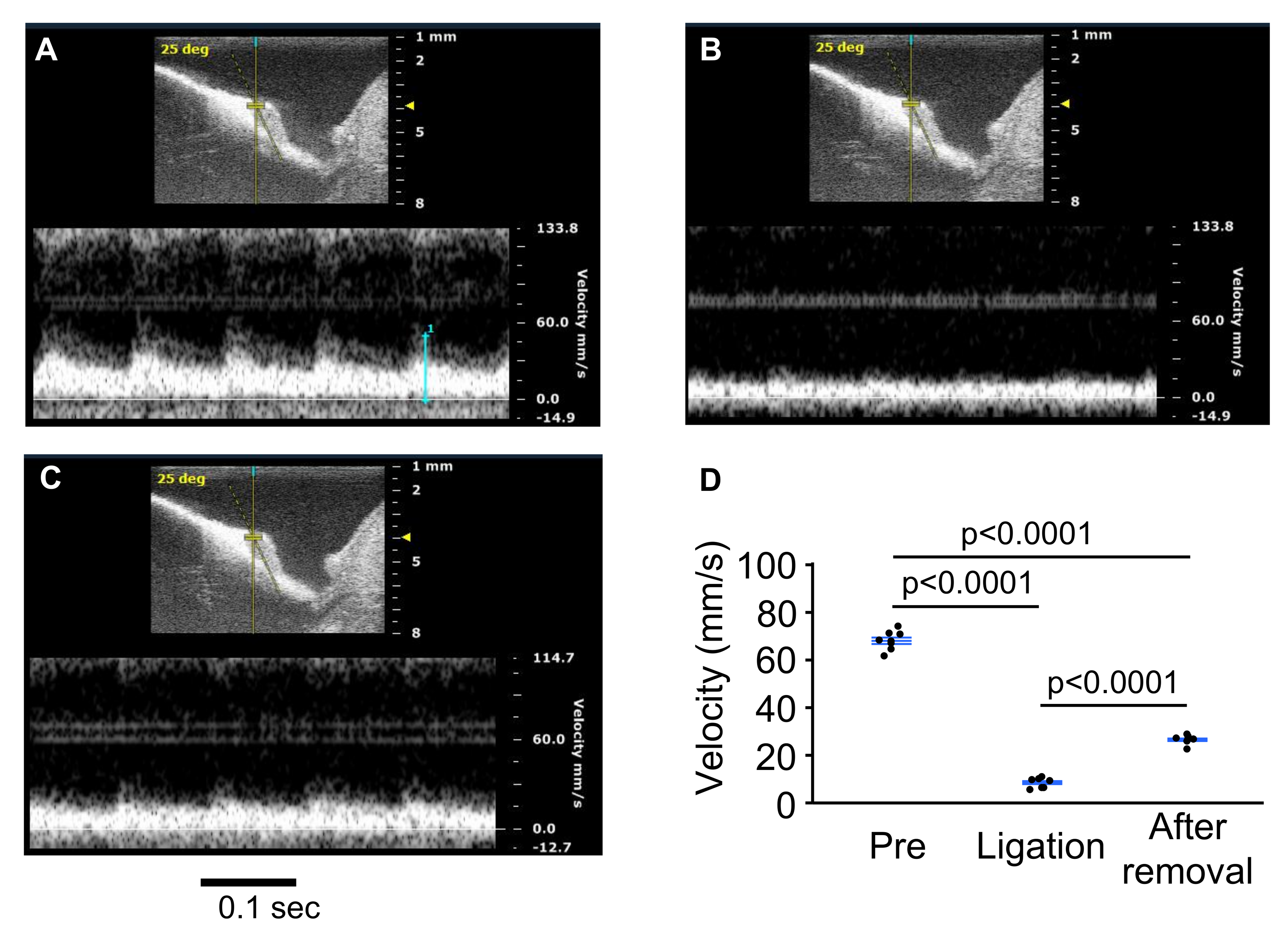

Supplement: S1 Fig — Doppler blood velocity waveforms were obtained from a uterine artery before (A) and after (B) ligation of uterine vessels with a nylon thread. (C) Doppler blood velocity waveforms after removal of the nylon thread. (D) Summary of arterial blood flow velocity of 2 uterine arcades (4 peaks each) before ligation, after ligation, and after removal of a nylon thread of uterine vessels. (TIF) [file pone.0155426.s001.tif]

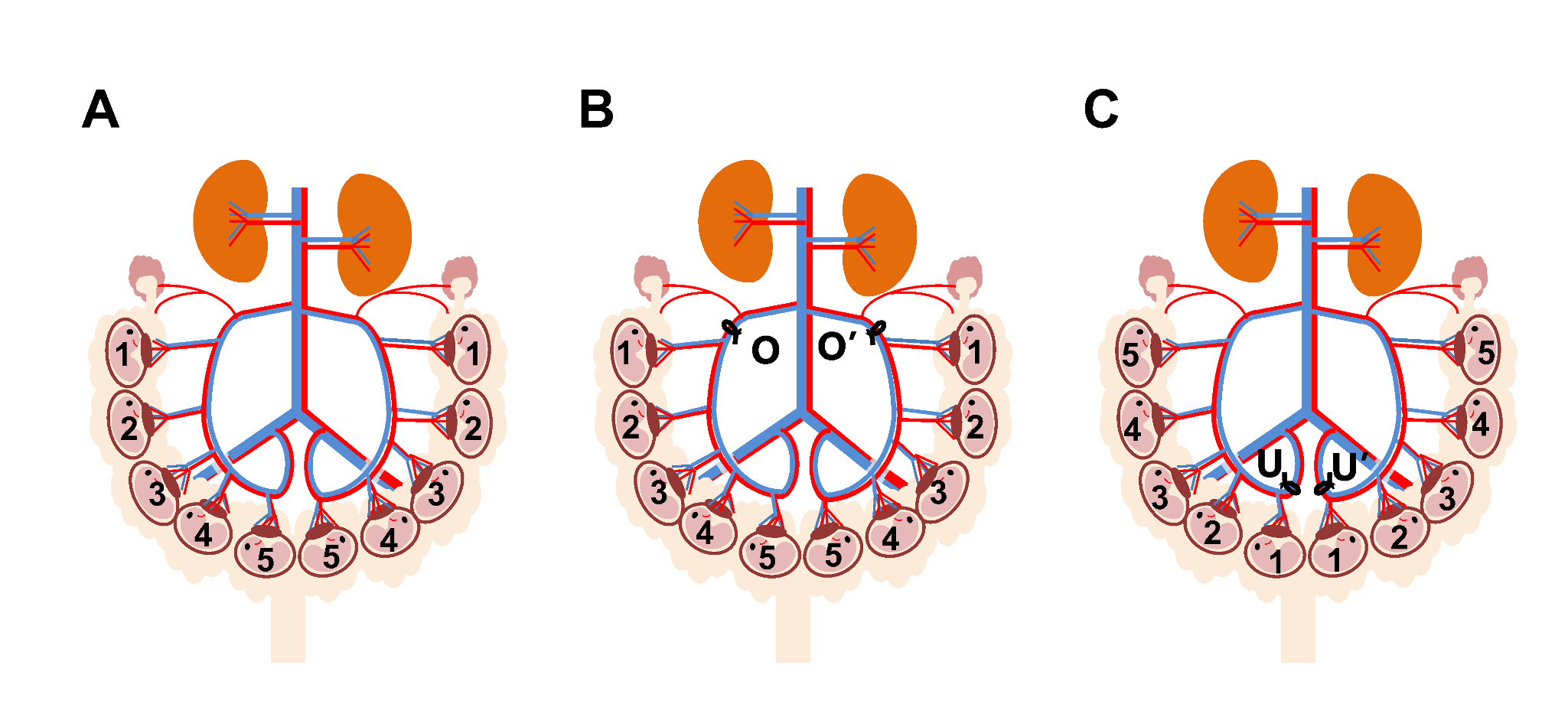

Supplement: S2 Fig — (A) sham mice, (B) O,O’ mice, (C) U,U’ mice. The number of the embryos were counted from the one closest to the ovaries in sham (panel A) or from the ligation position (panels B and C), when the pregnant mice have 5 embryos in right and left uterine horns each. In reality the number of embryos in each horn is from zero to 9 in ICR strain of mice. (TIF) [file pone.0155426.s002.tif]
