# Supplementary material for: Reduced Uterine Perfusion Pressure (RUPP) Model of Preeclampsia in Mice
Source: PLoS One. 2016 May 17;11(5):e0155426. doi: 10.1371/journal.pone.0155426 (PMC4871336; doi:10.1371/journal.pone.0155426)
Supplement: S1 Table — ap < 0.05 vs. sham, bp < 0.05 vs. O,O’, cp < 0.05 vs. U,U’. Data are expressed as means ± standard error of the mean. Values in parentheses show body weight and the number of dams without miscarriages at 18.5 dpc. (PDF) [file pone.0155426.s003.pdf]

	Sham	O,O’	U,U’	O,O’+U,U’
	n = 13	n = 14	n = 14	n = 11
BW (g) @ 0.5 dpc	31.8 ± 0.8	30.9 ± 0.6	32.1 ± 0.8	30.6 ± 1.1
BW (g) @ 14.5 dpc	47.2 ± 1.3	48.1 ± 1.2	49.5 ± 1.2	47.5 ± 1.9
BW (g) @18.5 dpc	59.5 ± 1.6	53.6 ± 2.2	44.2 ± 2.6 [a]	35.9 ± 3.1[a,b,c]
	(59.5 ± 1.6, 13)	(56.9 ± 2.0, 12)	(54.2 ± 1.1, 7)	(44.3, 1)
kidney (g)	0.19 ± 0.01	0.20 ± 0.01	0.22 ± 0.01	0.22 ± 0.01
Liver (g)	2.42 ± 0.07	2.52 ± 0.09	2.39 ± 0.16	1.78 ± 0.13[a,b,c]
Spleen (g)	0.15 ± 0.02	0.15 ± 0.02	0.20 ± 0.03	0.27 ± 0.05
Heart (g)	0.14 ± 0.02	0.17 ± 0.03[a]	0.19 ± 0.02[a]	0.18 ± 0.02[a]
Uterus (g)	23.6 ± 2.0	17.1 ± 1.9	8.8 ± 1.8[a,b]	3.7 ± 1.2[a,b]
