# Supplementary material for: Reduced Uterine Perfusion Pressure (RUPP) Model of Preeclampsia in Mice
Source: PLoS One. 2016 May 17;11(5):e0155426. doi: 10.1371/journal.pone.0155426 (PMC4871336; doi:10.1371/journal.pone.0155426)
Supplement: S2 Table — ap < 0.05 vs. sham, bp < 0.05 vs. O,O’, cp < 0.05 vs. U,U’, dembryonic weight less than tenth percentile of sham. Data are expressed as means ± standard error of the mean. FGR, fetal growth restriction. (PDF) [file pone.0155426.s004.pdf]

	Sham	O,O’	U,U’	O,O’+U,U’
	n = 13	*n* = 14	*n* = 14	*n* = 11
Litter size @14.5 dpc	13.8 ± 0.9	14.7 ± 0.8	15.7 ± 0.9	14.2 ± 1.3
Litter size @18.5 dpc	13.2 ± 1.0	8.7 ± 1.3[a]	5.4 ± 1.3[a]	1.2 ± 1.8[a,b,c]
No. of resorptions	0.5 ± 1.0	4.6 ± 0.9[a]	8.7 ± 1.3[a]	4.6 ± 2.2[a]
Embryo survival (%)	98.1 ± 1.1	60.8 ± 8.6[a]	37.2 ± 9.6[a,b]	3.5 ± 3.5[a,b,c]
Pregnancy with embryos (%)	100	84.6	46.7[a,b]	9.1[a,b,c]
FGR (%)[d]	9.7 ± 3.3	37.0 ± 10.1	58.9 ± 9.7[a]	100[a,b]
